# Nanomaterials-Based Electrochemiluminescence Biosensors for Food Analysis: Recent Developments and Future Directions

**DOI:** 10.3390/bios12111046

**Published:** 2022-11-18

**Authors:** Jiaojiao Zhou, Xuqin Lv, Jilai Jia, Zia-ud Din, Shiqi Cai, Jiangling He, Fang Xie, Jie Cai

**Affiliations:** 1 National R&D Center for Se-Rich Agricultural Products Processing, Hubei Engineering Research Center for Deep Processing of Green Se-Rich Agricultural Products, School of Modern Industry for Selenium Science and Engineering, Wuhan Polytechnic University, Wuhan 430023, China; 2 Key Laboratory for Deep Processing of Major Grain and Oil, Ministry of Education, Hubei Key Laboratory for Processing and Transformation of Agricultural Products, Wuhan Polytechnic University, Wuhan 430023, China; 3 Department of Agriculture, University of Swabi, Swabi 23561, Pakistan

**Keywords:** electrochemiluminescence, food analysis, biosensors, nanomaterials

## Abstract

Developing robust and sensitive food safety detection methods is important for human health. Electrochemiluminescence (ECL) is a powerful analytical technique for complete separation of input source (electricity) and output signal (light), thereby significantly reducing background ECL signal. ECL biosensors have attracted considerable attention owing to their high sensitivity and wide dynamic range in food safety detection. In this review, we introduce the principles of ECL biosensors and common ECL luminophores, as well as the latest applications of ECL biosensors in food analysis. Further, novel nanomaterial assembly strategies have been progressively incorporated into the design of ECL biosensors, and by demonstrating some representative works, we summarize the development status of ECL biosensors in detection of mycotoxins, heavy metal ions, antibiotics, pesticide residues, foodborne pathogens, and other illegal additives. Finally, the current challenges faced by ECL biosensors are outlined and the future directions for advancing ECL research are presented.

## 1. Introduction

Electrochemiluminescence (ECL), also called electrogenerated chemiluminescence, is a process where species generated at electrodes undergo high-energy electron-transfer reactions to generate electronically excited species that emit light [1,2]. Due to its linkage of electrochemistry and spectroscopy, this technique is superior to other optical methods, including photoluminescence, electrochemistry, and chemiluminescence [3,4]. Moreover, it features other advantages. First, it does not require an external light source, which not only simplifies detection instruments, but also reduces background signals, thus enabling high sensitivity [5]. Second, ECL is more selective than chemiluminescence due to its excellent controllability of light emission time and position by changing the applied potential [6], leading to greater selectivity, simplicity, and reproducibility, thereby facilitating the simultaneous measurement of multi-analytes [7,8]. Third, in several cases, ECL emitters can be regenerated after emission, allowing their further participation in ECL reactions, enabling the regeneration of numerous photons, thus increasing ECL sensitivity [9]. The above-mentioned features suggest the necessity for further development of ECL biosensors for trace target detection [10,11]. 

Recently, ECL has become a powerful analytical tool and is widely applied in many fields. Food safety is an international concern, and unsafe food can cause a variety of diseases and some of them can even be fatal [12]. Food safety issues are generally caused by the consumption of contaminated foods, due to abuse of pesticides, residue of antibiotics, heavy metal accumulation, and production of toxins. Furthermore, globalization further increases the complications of food analysis. These challenges suggest the importance of improving food analysis methods and detection techniques for monitoring food contamination. Over the past decades, various analytical methods have been developed for qualitative/quantitative food analysis [13,14,15,16]. On account of their fast analysis speed, simplicity, low cost, inexpensive equipment, and no background interference, ECL-based biosensors are attracting increasing attention in food analysis.

Despite its many fascinating advantages, the performance of ECL technology needs to be further improved to meet the increasing detection requirements. For example, some biosensors cannot meet the detection limits in practical applications. Fortunately, the development and improvement of nanotechnology have offered potential solutions to this problem, i.e., the flexible controllability of a nanomaterial endows it with a variety of application scenarios. A biosensor composed of nanomaterials and biomaterials can incorporate the excellent properties of nanomaterials and the binding affinity of biomaterials, making it an ideal choice to meet the growing needs of food safety analysis. Nanomaterials with different sizes, morphologies, and chemical components have been adopted in different biosensing applications [17]. In ECL-based biosensors, nanomaterials are multifunctional and play several different roles, such as improving the efficiency of both biological recognition and signal transducer. For example, certain kinds of nanomaterials with an enormous specific surface area can be used for immobilizing biomaterials and carrying ECL emitters. This indicates that nanomaterials-based ECL biosensors have great application potential in the detection of a diverse range of food contaminants.

**Figure 1:**
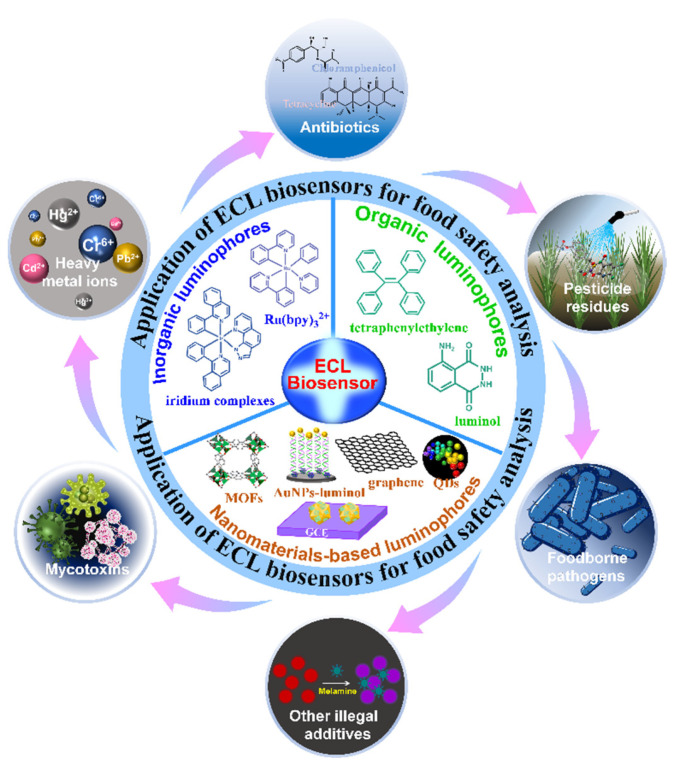
Scheme of the principles of ECL biosensors, ECL luminophores, and their ECL applications in food analysis.

Several excellent review papers have been published on nanomaterials-based ECL biosensors for food analysis [3,18,19,20,21]. However, they are mainly focused on ECL emitters or research published before 2020, and have not discussed emerging nanomaterials, such as Mxene and sensing strategies, nor the recent advances in nanomaterials-based ECL biosensors in food analysis. Therefore, this review aimed to give a succinct overview of nanomaterials-based ECL biosensors reported in the past few years. Specifically, we present the fundamentals of ECL biosensors and ECL luminophores, with a focus on the recent developments of ECL biosensors for food safety analysis, including analysis related to mycotoxins, heavy metal ions, antibiotics, pesticide residues, foodborne pathogens, and other detrimental and/or prohibited additives (Figure 1). Additionally, more biosensing strategies are proposed to facilitate the creation of portable devices for food analysis. 

## 2. Fundamentals of ECL Biosensors

### 2.1. Composition of ECL Biosensors

An ECL biosensor is primarily composed of an electrochemical workstation, photomultiplier, and signal acquisition system, while a general instrumentation for ECL research mainly includes two components: an electrochemical workstation and an optical device. In both cases, light is acquired through a photomultiplier tube. Consistent with this energy conversion process, ECL luminophores, including inorganic luminophores, organic luminophores, and nanomaterials-based luminophores, provide excellent signal transduction pathways for ECL-based analysis. 

### 2.2. Mechanism of ECL

The first ECL studies were performed separately by Hercules and Bard [22,23]. Since then, efforts have been made to investigate the mechanism of ECL. Based on the source of radicals, the mechanism can be divided into an annihilation mechanism and coreactant mechanism. In the annihilation pathway, both oxidized and reduced species are produced during a potential sweep, where radical cations (A^•+^) and anions (A^•−^) interact with each other to produce a ground state (A) and an excited state A*. Then, the excited state A* relaxes to the ground state A, leading to energy release through light emission. Despite no requirement of additional reagents in the annihilation process, the ECL species must be chemically stable and maintain sufficient charge states. Moreover, a sufficient potential is required for production of oppositely charged species. 

Because of these limitations, the coreactant pathway is more commonly used for ECL analysis. In the coreactant pathway, ECL is typically produced by a potential sweep [2], where coreactant radicals react with luminophore radicals to generate excited states, which subsequently emit light upon returning to the ground state. The coreactant pathway can overcome the above-mentioned limitations, enabling more efficient and sensitive ECL in the presence of coreactants. The widely used coreactants include hydrogen peroxide (H_2_O_2_), peroxydisulfate (S_2_O_8_^2−^), O_2_, tri-n-propylamine (TPrA), and oxalate (C_2_O_4_^2−^). With a reductant or oxidant coreactant, coreactant ECL can be divided into two categories: oxidative reduction and reductive oxidation, with the former occurring at positive potentials, and the latter at negative potentials. Currently, ECL coreactants have drawn considerable attention due to the dominant role of this mechanism in this field.

### 2.3. ECL Biosensing Strategies

**Figure 2:**
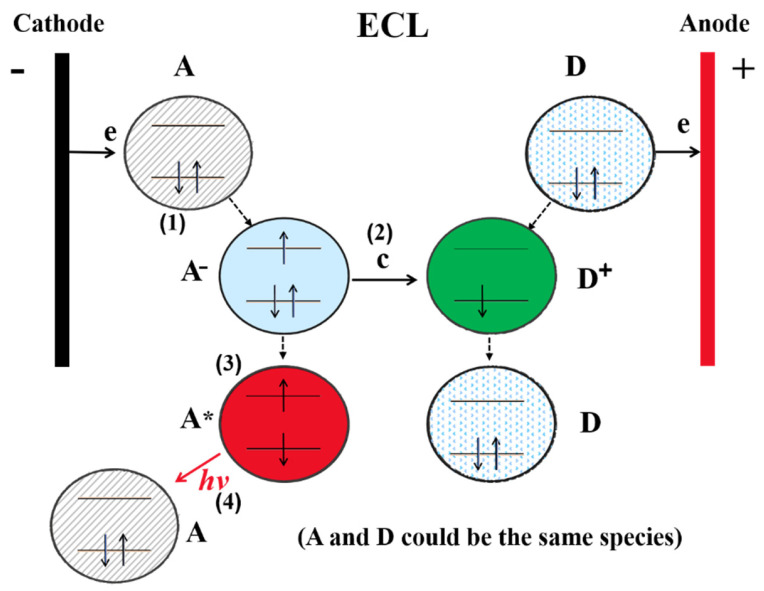
Scheme of ECL process. Reproduced with permission from Ref. [24]. Copyright 2017, Elsevier.

ECL biosensing is based on the detection of interactions between specific recognition elements and targets through ECL emission changes, with the involvement of two critical components: recognition molecules and luminophores. Common luminophores include organic and inorganic materials, as well as nanomaterials. Generally, most ECL systems comprise four processes (Figure 2) [24]: (1) redox reactions, (2) homogeneous chemical reactions, (3) excited-state formation, and (4) light emission. Any substances that can affect the ECL process will change the ECL emission and allow it to be detected quantitatively.

ECL biosensing strategies are typically of five types [24]. In the first strategy, the ECL luminophore is used as a signal tag. Another common strategy is based on steric hindrance from the recognition reaction, which is suitable for biomacromolecule detection, and this type of biosensor follows the basic principles of a biorecognition reaction, where the ECL signal is immensely hindered, generating a simple and standard system capable of detecting a sample with high sensitivity and selectivity. The third strategy is through the interaction between targets and ECL emitters, where any target that can affect the ECL characteristics will change ECL emission, allowing this phenomenon to be used for target analysis, i.e., targets will preferably quench the ECL signal by altering the working surfaces of luminophores, leading to a change in ECL signal, which is related to the analyte concentration. The fourth strategy is based on the interaction between analytes and coreactants, where any substance that can react with the coreactant can be measured by the change in ECL, and this method is mainly used to detect the charge transmission between excited states and the quencher. The fifth strategy is referred to as ECL resonance energy transfer (ECL-RET), where the emission spectrum of a donor overlaps with the absorption spectrum of an acceptor, i.e., a nonradiative process with transmission of energy occurring between an excited species donor and a ground species recipient. 

## 3. ECL Luminophores

### 3.1. Inorganic Luminophores

Ru(bpy)_3_^2+^, the first reported inorganic luminophore, has been widely used because of its excellent ECL properties and high stability. The ECL properties of Pt(II) and Ir(III) have recently been investigated [25,26]. Notably, by changing their molecular structures, various cyclometalated complexes can overcome the shortcomings of narrow selection of ECL emitters. For instance, Guo et al. reported that several ruthenium and iridium complexes could exhibit distinguishable ECL signals [26]. Moreover, Carrara et al. were the first to report that square-planar Pt(II) complexes possess aggregation-induced ECL (AIE-ECL) [25]. Essentially, by adjusting the solvent, square-planar Pt(II) complexes can aggregate into supramolecular systems and exhibit enhanced ECL intensity, which can be attributed to strong intermolecular Pt–Pt interactions, enabling new ECL pathways to produce excited states. Later, Gao et al. investigated the highly efficient AIE-ECL of cyclometalated iridium(III) complexes in aqueous solution [27]. These molecules are connected through π–π stacking interactions and hydrogen bonds, thus facilitating their self-assembly into nanoparticles. More importantly, such nanostructures exhibit a multifold increase in ECL intensity, thus holding a great promise for practical applications.

### 3.2. Organic Luminophores

Over the past several years, polyaromatic hydrocarbons (PAHs) have been thoroughly investigated for ECL applications owing to their low cost, high quantum yield, and superior structural tailorability [28]. Additionally, these compounds have been extensively investigated in organic systems, including luminols and organic molecules, which considerably promoted the development of material science, and readers can refer to several previous ECL reviews [1,9,29,30] for more information on organic systems. 

Luminol has long been a common organic luminophore owing to its low cost, easy functionalization, and high ECL efficiency. However, its low solubility under physiological conditions considerably affects its practical applications, leading to the development of new luminophore derivatives. For example, Baeumner et al. generated a water-soluble luminol by introducing a carboxylate group, resulting in a multifold increase in ECL signal relative to luminol [31]. Cathodic luminol ECL is usually very weak when luminol is not oxidized on the electrode at negative potentials, and anodic luminol ECL generally requires the addition of hydrogen peroxide. 

Organic molecules with AIE-ECL properties have been continuously investigated. For example, Yuan et al. reported that aggregated tetraphenylethylene (TPE) microcrystals exhibit a strong ECL intensity because of the restriction of intramolecular motion-driven ECL enhancement [28]. Lu et al. reported the detailed ECL mechanism of TPE [32], and they found that electron-withdrawing nitro-substituted TPE exhibits a stronger ECL behavior owing to a reduced LUMO/HOMO bandgap.

### 3.3. Nanomaterials-Based Luminophores

Since the first ECL study of silicon nanocrystal QDs [33], a series of QDs and corresponding alloyed or core–shell-structured QDs have been reported. Nanomaterials play a significant role in ECL research by essentially functioning as emitters, accelerators, carriers, and multifunctional nanocomponents. In this section, we briefly introduce nanomaterials-based luminophores for sensing applications.

Noble metal nanoclusters (NCs), another type of ECL emitter, are favored for their ultrasmall size, facile preparation, low toxicity, and good biocompatibility. For example, water-soluble AuNCs exhibit enhanced ECL emission because of the formation of a rigid shell through a host–guest interaction on the AuNC surface [34]. In another study, this same group reported dual enhancement of AuNC ECL with a combination of electrocatalytic excitation and aggregation-induced emission using 6-aza-2-thiothymine as the matrix [35]. Bimetallic NCs have attracted significant attention compared with monometallic NCs because of the synergetic effects of the two atoms. For instance, Au–Ag bimetallic NCs could achieve multifold enhanced signals relative to NCs based on individual metals [36].

Nanoscale metal oxides are another type of ECL emitter. For example, Wei et al. explored the ECL properties of CeO_2_ nanoparticles with K_2_S_2_O_8_ as the coreactant [37,38], and due to relatively low electron conductivity, graphene oxide (GO) and AuNPs were used as carriers of CeO_2_ to increase its conductivity. Compared with undoped graphene, nitrogen-doped graphene decorated with ZnO nanocrystals exhibits a high ECL intensity [39], allowing the immobilization of ZnO nanocrystals on nitrogen-doped graphene to achieve a multifold increase in ECL intensity and a shift in the onset ECL potential.

Organic NPs with ECL properties can also be used as emitters. For instance, Luo et al. prepared poly(9,9-dioctylfluorenyl-2,7-diyl) dots with improved ECL properties without the assistance of any coreactants [40]. Organic NPs coupled with donor–acceptor components can be used to design ECL-RET biosensors. 

Meanwhile, many other synthetic materials are also used as ECL luminophores, such as metal–organic frameworks (MOFs) and covalent organic frameworks (COFs). Both MOFs and COFs are porous crystalline framework materials that are amenable to design and functionalization. In combination with ECL luminophores as organic linkers, MOFs and COFs are two typical representatives of the new generation of ECL emitters [41,42]. 

Furthermore, nanomaterials can act as nanocarriers of luminophores for signal amplification, with popular nanocarriers including metal nanoparticles (particularly AuNPs and AgNPs), magnetic nanoparticles, two-dimensional nanomaterials (such as GO, MXene, and metal–organic layers), nanofibers, nanorods, and silica NPs. Sensing platforms based on these nanocomposites exhibit amplified ECL intensities and high sensitivity toward analytes. 

## 4. Applications of ECL Biosensors in Food Analysis 

### 4.1. Mycotoxins

Mycotoxins are the secondary metabolites produced by some fungi that can cause severe side effects in organisms [43]. Moreover, multiple mycotoxins can coexist within a single food matrix, with common mycotoxins including aflatoxin B1 (AFB1), ochratoxin A (OTA), deoxynivalenol (DON), and zearalenone (ZON) [44,45]. Among them, AFB1 and OTA are the most toxic. Over the past several decades, great efforts have been devoted to the development of biosensors for mycotoxin detection, and some such ECL biosensors are summarized in Table 1.

AFB1 is the most toxic mycotoxin because of its serious side effects [46]. Based on the competitive binding between AFB1–bovine serum albumin and free AFB1 with antibody–AFB1, Wang et al. fabricated a competitive ECL immunosensor for AFB1 detection [47], with AuNP-modified Ru(bpy)_3_^2+^ as an emitter and also as a matrix for antibody–AFB1 immobilization through Au–NH_2_. Under optimal conditions, the proposed immunosensor exhibited a good linear range from 0.01 to 100 ng/mL with a detection limit of 0.0039 ng/mL (S/N = 3). To improve the sensitivity of AFB1 assays, Yan et al. designed an enhanced ECL aptasensor for AFB1 detection using enzyme-assisted 3D DNA nanoflowers for signal amplification [48], and this aptasensor can achieve the quantification of AFB1 with a good linear range from 1 pg/mL to 5 ng/mL with a detection limit of 0.27 pg/mL due to the synergistic effects between the competitive reaction and enzyme-assisted amplification strategy.

**Figure 3:**
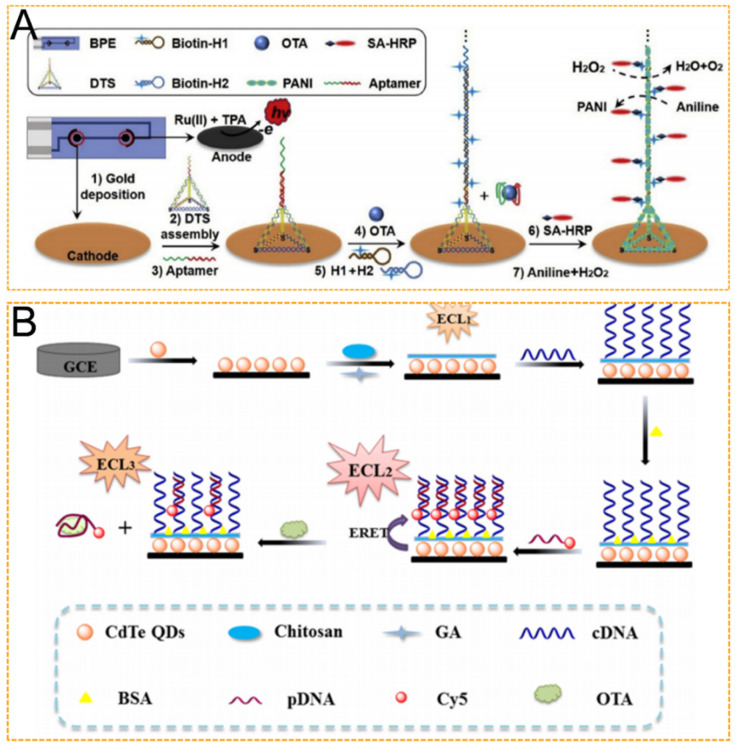
(**A**) Scheme of bipolar electrode ECL biosensor for OTA detection. Reproduced with permission from Ref. [48]. Copyright 2021, Elsevier. (**B**) Scheme of ECL aptasensor for OTA detection using a DNA walking machine. Reproduced with permission from Ref. [50]. Copyright 2019, Elsevier.

Ochratoxin A (OTA) is another toxic and widespread mycotoxin commonly found in cereals, coffee, and wine [49]. To improve OTA assay accuracy, Lu et al. designed a three-step strategy for OTA detection based on a bipolar electrode (BPE) ECL aptasensor [50] (Figure 3A). Firstly, the single strand probe is replaced by a DNA tetrahedron-structured aptamer (DTA). Secondly, a large number of negatively charged nucleic acid backbones are provided by hybrid chain reaction (HCR) and DTA as templates to form polyaniline (PANI) deposition. Thirdly, the biosensor uses a cathode of closed BPE as a functional sensing interface and an anode as a signal collection interface. The analyte does not participate in the ECL reaction, thus avoiding direct contact of photoactive molecules, greatly reducing the effect of the food matrix on signal acquisition, and achieving a detection limit of 3 pg/mL. Compared with three-electrode biosensors and high-performance liquid chromatography (HPLC) analysis, this BPE strategy simplifies pretreatment methods and improves detection accuracy. In another study, by combining ECL-RET and DNA nanotechnology, Wei et al. devised a sensitive ECL aptasensor for OTA assays [51] (Figure 3B). First, Cy5 quenched the ECL intensity of CdS QDs, and OTA addition triggered hybridization between the walker and Cy5-labeled DNA, followed by the release of Cy5-DNA with the assistance of a nicking endonuclease, thereby resulting in the recovery of the ECL signal of CdS QDs. This aptasensor achieved a linear range from 0.05 nM to 5 nM with a detection limit of 0.012 nM for OTA detection due to the introduction of the DNA walking machine and the excellent properties of the ECL technique.

Mycotoxins such as deoxynivalenol (DON), FB1, and ZEN can contaminate a wide range of agricultural products, including wheat, flour, milk, and corn [52,53]. To ensure better sensitivity, Luo et al. devised a self-enhanced ECL aptasensor for ZEN analysis based on Ru(bpy)_3_^2+^-doped silica nanoparticles (Ru@SiO_2_ NPs) and nitrogen-doped graphene QDs (NGQDs) [54]. Essentially, the combination of these two nanomaterials shortened the electron-transfer distance between the emitter (Ru(bpy)_3_^2+^) and the coreactant (NGQDs), thus enhancing the sensitivity for ZEN measurement in food samples. This self-enhanced ECL aptasensor expressed the widest linear range from 10 fg/mL to 10 ng/mL and the lowest detection limit of 1 fg/mL for ZEN detection. The selectivity can also be improved through the combination of immunoassays. By using NiFe_2_O_4_ nanotubes modified with Ru(bpy)_3_^2+^ as ECL probes to generate enhanced ECL signals, and TiO_2_ mesocrystals as the sensing platform and support for antibody immobilization, Fang et al. developed a competitive ECL immunoassay for ZEN measurement with a wide linear range of 0.1–10 ng/mL with a detection limit as low as 3.3 fg/mL [55]. Therefore, this immunosensor is capable of quantitatively detecting ZEN and has high sensitivity.

**Table 1:** Brief summary of ECL biosensors for the mycotoxin sensing in food samples.

Analytes	Sample Matrix	Limit of Detection	Linear Range	Ref.
Aflatoxin B1	peanut	0.17 ng/mL	3.13–125.00 ng/mL	[46]
Aflatoxin B1	milk	3.9 pg/mL	0.01–100 ng/mL	[47]
Aflatoxin B1	peanut, wheat	0.27 pg/mL	1 pg/mL–5 ng/mL	[48]
Ochratoxin A	corn	0.17 pg/mL	0.0005–50 ng/mL	[49]
Ochratoxin A	grain	3 pg/mL	0.01–500 ng/mL	[50]
Ochratoxin A	wine, beer	0.012 nM	0.05 nM–5 nM	[51]
Zearalenone	corn flour	1 fg/mL	10 fg/mL–10 ng/mL	[54]
Zearalenone	coconut milk	3.3 fg/mL	10 fg/mL–0.1 ng/mL	[55]

### 4.2. Heavy Metal Ions

Heavy metal ions are non-biodegradable and can cause environmental pollution, thus considerably affecting human health. Common heavy metals, such as mercury (Hg), lead (Pb), and chromium (Cr), are toxic, and current ECL biosensors for heavy metal ion sensing are summarized in Table 2.

**Figure 4:**
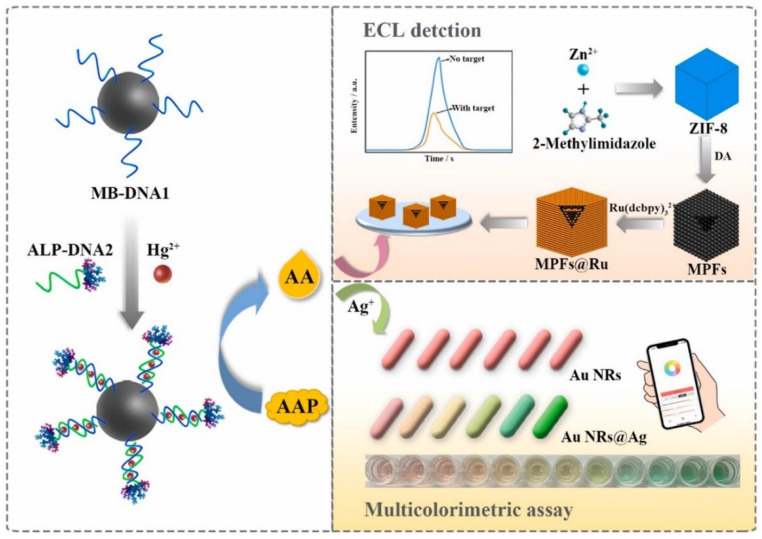
Scheme of the ECL and colorimetric sensor for Hg^2+^ detection. Reproduced with permission from Ref. [57]. Copyright 2021, Elsevier.

Mercury is one of the most toxic elements affecting the health of humans and ecosystems [56]. For Hg^2+^ detection, Ma et al. developed a novel dual-modal sensor with T-rich DNA probes based on T–Hg^2+^–T configuration [57] (Figure 4). Briefly, magnetic bead-linked DNA1 was bound with alkaline phosphatase-labeled DNA2 to form T–Hg^2+^–T structures upon Hg^2+^ addition. Subsequently, the catalytic product (ascorbic acid) quenched the ECL intensity of (Ru(dcbpy)_3_^2+^) and stimulated the deposition of silver shells on the gold nanorods, thus resulting in a series of multicolor variations. Under optimized conditions, the dual-modal assay showed an excellent response for Hg^2+^ assays in the linear range of 2 pM to 500 nM with a low detection limit of 0.32 pM for the ECL method. The recovery of lake water samples ranged from 98.53% to 111.97%, revealing prospective applications in monitoring environmental water samples. Additionally, Ma et al. reported a ‘‘turn-off’’ ECL sensor for Hg^2+^ detection based on T–Hg^2+^–T configuration with a DNA three-way junction structure tagged with a ruthenium(II) complex [58], where the confirmation of a DNA three-way junction allowed ruthenium(II) to be away from the electrode upon Hg^2+^ addition, leading to a decrease in ECL signal, and a linear relationship was obtained in the range of 0.1–10 pM with a detection limit of 0.04 pM. 

Lead ions (Pb^2+^) are common toxic and persistent contaminants that can cause severe health risks owing to their toxicity, perdurability, and bioaccumulative nature [59], and exposure of humans to Pb^2+^ can cause severe damage to the brain, bones, kidneys, liver, immune system, and nervous system [60]. As a near-infrared ECL luminophore coupled with a Pb^2+^-dependent DNAzyme and DNA amplification strategy, porphyrin dots have been used to establish a “signal-on” ECL biosensor for Pb^2+^ detection [61]. A dynamic range from 10 pM to 1 μM with a low limit of detection (LOD) of 1.2 pM is obtained. Meanwhile, this ECL biosensor also serves as an example of organics-based dot luminophores for ECL research. Due to the advantages of low cost, simplicity, and portability, the development of paper-based chips has attracted increasing attention. For example, an integrated lab-on-paper device has been prepared by using reduced graphene oxide (rGO)–PdAu–glucose oxidase nanocomposites, where the Pb^2+^-specific DNAzyme was hybridized with oligonucleotides labeled with these nanocomposites, triggering the release of the labeled probe by adding Pb^2+^, owing to the specific binding between Pb^2+^ and DNAzyme, thereby promoting oxidation of 3,3′,5,5′-tetramethylbenzidine (TMB). Moreover, the H_2_O_2_ produced by the enzymatic reaction is a coreactant for luminol in the ECL reaction, thus enabling the monitoring of the decreased ECL intensity of luminol in a linear range of 0.5–2000 nM for Pb^2+^ sensing [62]. Additionally, Wang et al. designed an ECL-RET platform for sensitive and selective detection of Pb^2+^. In particular, the ECL emission spectrum of two-dimensional black phosphorus overlapped with the absorption spectrum of Ag/AgCl nanocubes, and the presence of Pb^2+^ resulted in the desorption of Ag/AgCl nanocubes to induce enhanced ECL emission, enabling this assay method to accurately quantify Pb^2+^ in the range between 0.5 pM and 5 nM with an LOD of 0.27 pM [63]. Moreover, based on a flexible switching conformation from “open” to “closed”, Zhao et al. designed an “on–off” ECL biosensor using a DNA nanotweezer for a Pb^2+^ assay [64], where the introduction of Pb^2+^ induced the DNA nanotweezer to switch from “open” to “closed,” which could be used to capture abundant hemin in the closed state, leading to a decrease in the ECL intensity of Ru(phen)_3_^2+^. There was a good linear relationship between the changes in ECL signal and concentration of Pb^2+^ over a wide range from 10 fM to 10 nM with an LOD of 1.9 fM. This method provides a universal ECL biosensor for assaying heavy metal ions due to the structural flexibility of DNA tweezers.

Hexavalent chromium (Cr(VI)) is a highly toxic contaminant that can cause numerous adverse environmental effects and pose serious threats to human health [65,66]. Guo et al. successfully designed tetraphenylbenzosilole derivatives as ECL luminophores to generate an aggregation-induced ECL (AIECL) sensor for Cr(VI) detection [67]. The ECL sensor exhibited excellent detection performance for Cr(VI) with a wide linear range from 10^−12^ to 10^−4^ M and an extremely low detection limit of 0.83 pM.

Furthermore, there are also reports on multiple determinations of heavy metals by ECL biosensors. Moghaddam et al. developed a bipolar ECL sensor for multiple determinations of heavy metals [68], where the color of the bipolar ECL emission not only depended on the identity of heavy metals but also on their concentration, achieving both qualitative and quantitative analyses of heavy metals. This sensor exhibited a detection limit of 0.094 μM and 0.008 μM for Cd^2+^ and Cu^2+^, respectively.

**Table 2:** Brief summary of ECL biosensors for heavy metal ion sensing in water.

Analytes	Sample Matrix	Limit of Detection	Linear Range	Ref.
Cr(VI)	lake water	0.83 pM	10 pM–0.1 mM	[67]
Hg^2+^	lake water	0.32 pM	2 pM–500 nM	[57]
Hg^2+^	water	0.04 pM	0.1–10 pM	[58]
Pb^2+^	tap water, lake water	0.27 pM	0.5 pM–5 nM	[63]
Pb^2+^	water	1.9 fM	10 fM–10 nM.	[64]
Pb^2+^	drinkable water	1.2 pM	10 pM–1 μM	[61]
Pb^2+^	tap water, river water	0.14 nM	0.5–2000 nM	[62]
Cd^2+^ and Cu^2+^	-	Cd^2+^: 0.094 μM Cu^2+^: 0.008 μM	Cd^2+^: 1 μM–75 μM, Cu^2+^: 0.1–1.75 μM	[68]

### 4.3. Antibiotics

Antibiotics are extensively used in aquaculture and animal husbandry. However, the overuse of antibiotics can lead to adverse consequences, such as antimicrobial resistance and various side effects. Therefore, it is necessary to develop rapid and sensitive methods for the detection of antibiotics. Current ECL biosensors for antibiotic detection are summarized in Table 3.

The β-lactam antibiotic amoxicillin (AMX) is widely used in animal husbandry, and its residue accumulation in the environment, animal products, and other agricultural products poses a threat to human health. For AMX determination, Kamyabi et al. constructed an ECL sensing platform based on the sensitive reaction between AMX and luminol, which exhibited high sensitivity toward amoxicillin in the wide linear range of 40 pM–65 μM with a low detection limit of 8.3 pM and a relative standard deviation of 1.42% [69]. Additionally, Li et al. reported a novel MIP sensor with GO loaded with CdTe QDs/Au NPs as an efficient ECL luminophore for AMX analysis, thus establishing a signal on/off switching sensor based on the ability of AMX to quench the ECL signal of GO/CdTe/Au NPs due to steric hindrance [70]. There was a wide linear range from 50 pM to 15 nM with a low detection limit of 8.3 pM for the detection of AMX.

Chloramphenicol (CAP) is widely used in animal husbandry, and residual CAP in food can adversely affect human health. For CAP detection, Li et al. designed an ECL immunoassay using hollow titanium dioxide and SnS_2_ QDs [71]. After modification with polyethyleneimine and gold nanoparticles, the dioxide hollow spheres could act as a coreaction accelerator to increase the ECL efficiency, achieving high sensitivity for CAP detection with a wide linear range of 0.01–100 ng/mL with an LOD of 3.1 pg/mL. 

**Figure 5:**
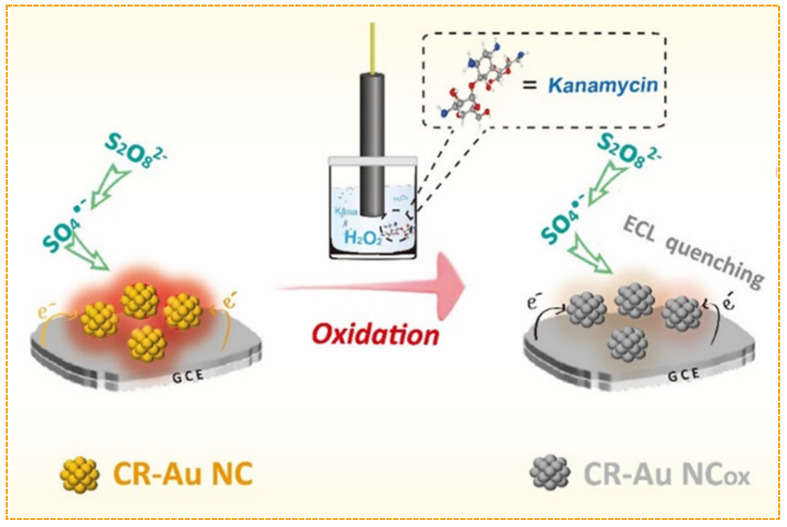
Scheme of the ECL sensor for kanamycin detection. Reproduced with permission from Ref. [73]. Copyright 2021, American Chemical Society.

The aminoglycoside antibiotic kanamycin is widely used to treat tuberculosis. For kanamycin analysis, Zhou et al. devised a fluorescent aptasensor based on synchronization signal amplification of a primer exchange reaction and metal-ion-dependent DNAzyme, which achieved a detection limit as low as 0.36 nM [72]. Additionally, Huang et al. reported an efficient ECL sensor for kanamycin detection using AuNCs as luminophores [73] (Figure 5), where Au0 could be oxidized to AuI through a redox reaction between kanamycin and AuNCs, leading to a decrease in ECL signal, and the as-fabricated ECL sensor exhibited a detection limit of 1.5 pM without using the signal amplification method.

Tetracycline is a broad-spectrum antibiotic, and its unwarranted application has resulted in many food safety problems that need to be solved. For tetracycline detection, Wang et al. devised a low-potential ECL immunoassay based on gold-filled photonic crystal electrodes, which achieved a tetracycline detection limit of 0.075 pg/mL [74]. Additionally, terbium(III) organic gels (TOGs) were prepared by mixing terbium ions and ligand, which could be used as ECL signal reporters for label-free detection of tetracycline [75]. The quenching mechanism of tetracycline was attributed to its reaction with the oxidizing sulfate radical, thus hindering the ECL process. The proposed method indicated high sensitivity toward tetracycline in the range of 0.1–25 μM with an LOD of 32.4 nM.

### 4.4. Pesticide Residues

Although their residues can cause serious problems to both the environment and human health, pesticides are still widely used in agriculture for insect eradication and crop protection, so it is necessary to develop methods for testing pesticide residues in food, water, and soil. 

Chlorpyrifos is an organophosphorus acetylcholinesterase inhibitor, and its overuse can cause serious environmental pollution. Based on an efficient ECL-RET strategy between MoS_2_/CdS nanospheres and silver/carbon QDs (Ag/CQDs), an ECL aptasensor was devised for chlorpyrifos detection with a detection limit of 0.35 fM [76]. Similarly, based on a ternary nanocomposite (ruthenium nanobeads/AgNPs/GO) acting as an efficient luminophore, an enzyme-free ECL platform has been generated for precise detection of chlorpyrifos [77]. A wide linear range and a low detection limit were obtained: from 5.0 aM to 4.2 nM and 0.65 aM, respectively.

Glyphosate is another organophosphorus compound used as a broad-spectrum herbicide. For glyphosate detection, a quenching-type ECL sensor has been developed by using luminol–AuNPs–L-cysteine–Cu(II) composites as ECL emitters and H_2_O_2_ generated by an enzymatic hydrolysis reaction as a coreactant [78]. In the presence of glyphosate, the acetylcholinesterase activity is inhibited, leading to the formation of a complex with Cu(II), causing Cu(II) to separate from the surface of the electrode and lose its catalytic effect. The constructed sensor could effectively enhance the selectivity of the enzyme inhibition sensor, achieving a detection limit of 0.5 nM for glyphosate.

Acetamiprid is an extensively used neonicotinoid for pest control in crops, and its improper use poses a threat to public health and environment. For acetamiprid detection, Guo et al. designed a Au-tetrahedral aptamer nanostructure that can catalyze the formation of H_2_O_2_ to enhance the ECL efficiency of luminol for acetamiprid detection [79]. Under the optimal conditions, the aptasensor had a detection limit of 0.0576 pM. In addition, by using luminol and graphite-like carbon nitride nanosheets (g-C_3_N_4_) as two potential-resolved ECL luminophores, a dual-signal ECL aptasensor was generated based on hollow Cu/Co–MOF–luminol and g-C_3_N_4_ for simultaneous detection of acetamiprid and malathion [80]. This ECL aptasensor for simultaneous detection of acetamiprid and malathion exhibited good sensitivity with a linear range from 0.1 μM to 0.1 pM, and detection limits of 0.015 pM and 0.018 pM (S/N = 3), respectively.

### 4.5. Foodborne Pathogens

*Vibrio,* a gram-negative bacterium widely present in food, can pose a potential threat to human health. For detection of *Vibrio parahaemolyticus* (*Vp*), a sensitive ECL biosensor has been devised based on [Ru(bpy)_2_(phen-5-NH_2_)]^2+^ as the signal reporter and AgNPs as the signal enhancer, and exhibited clear signal amplification effects for its unique Faraday cage structure and achieved a detection limit of 33 CFU/mL under optimal conditions [81]. In another study, a dual-mode ECL/SERS immunosensor was constructed for *Vibrio vulnificus* detection by using MXene material as a signal unit [82]. Owing to the large surface area of MXene, abundant signal tags are activated within the Faraday cage-type sensor, resulting in enhanced ECL signaling. Under optimal experimental conditions, the linear range and limit of quantification (LOQ) of ECL were from 1 to 10^8^ CFU/mL and 1 CFU/mL.

*Staphylococcus aureus* (*S. aureus*) is a major cause of bacterial food poisoning. For rapid detection of *S. aureus,* an enzyme-free ECL aptasensor was fabricated by using MoS_2_–PtNPs–vancomycin nanocomposites to decrease the ECL of the S_2_O_8_^2−^/O_2_ system [83]. The ECL intensity had a linear relationship with the logarithm of *S. aureus* concentration in a range of 1.5 × 10^2^ to 1.5 × 10^8^ CFU/mL, possessing the detection limit of 28 CFU/mL. Additionally, Liu et al. devised a novel universal signal amplification probe-based ECL biosensor for sensitive detection of *S. aureus* [84], where two distinct functional parts were carefully designed, with one part as the recognition region and the other as the reporter, thus enhancing the assay’s accuracy, specificity, and sensitivity. The experimental results showed that the detection limit was 100 fM.

### 4.6. Other Illegal Additives

Food additives are critical to the food industry because of their ability to improve food quality and facilitate food preservation. However, adding prohibited additives to food poses a serious threat to the environment and organisms. Therefore, it is necessary to develop sensitive and simple methods for monitoring detrimental or prohibited additives.

Residual environmental hormones can pose a risk to human and animal health through migration from packaging materials to food matrices, so the determination of environmental hormone levels is important for the national economy and people’s livelihoods. In the past few years, sensitive ECL biosensors have been developed for detecting environmental hormones from packaging materials. For instance, Huo et al. used CdSe QDs to modify working electrodes for ECL detection, enabling the modified electrodes to exhibit an LOD of 0.54 nM for bisphenol A and an LOD of 1.84 nM for dibutyl phthalate [85]. Additionally, Zhao et al. used CdTe@ZnS QDs to enhance the ECL intensity of a [Ru(bpy)_3_]^2+^–tripropylamine system through energy transfer and constructed a sandwich magnetically imprinted immunosensor for diethylstilbestrol (DES) detection based on the enhanced luminescence of Ru@SiO_2_ by CdTe@ZnS QDs [86]. Under the optimized conditions, the linear range was from 0.48 pM to 36 nM with an LOD of 0.025 pM. It is worth noting that the steroid hormone 17β-estradiol (E2) plays a crucial role in multiple organ functions, and abnormal E2 metabolism can disrupt endocrine function and cause severe internal organ injury, suggesting the importance of accurate E2 detection in physiological and environmental conditions. In this regard, Liu et al. designed an ECL-RET system for E2 detection based on α-FeOOH@CdS nanospheres as ECL nanoemitters and AgNCs as efficient quenchers [87], where the α-FeOOH nanospheres with a 3D hierarchical structure not only facilitated the immobilization of CdS QDs but also participated in a Fenton-like process. Additionally, AgNCs could quench the ECL signal of CdS QDs due to their well-matched donor–acceptor spectra for efficient energy transfer, thus endowing this ECL-RET biosensor with a wide detection range of 0.01–10 pg/mL with an LOD of 3 fg/mL.

## 5. Challenges and Perspectives

ECL biosensors integrate electrochemistry with spectroscopy, thus combining their merits of high sensitivity, simplicity, and rapidity. The key to the development of ECL biosensors is the synthesis of electrochemiluminescent materials with high efficiency, high stability, and good biocompatibility. The past decade has witnessed the development of ECL biosensors in food analysis, enabling ECL as a powerful tool for ultrasensitive detection of a wide range of analytes. Various strategies are developed to improve the efficiency of ECL assays, and thousands of relevant papers have been published in the past 5 years. The achievements of novel ECL luminophores have widened ECL sensing strategies, leading to the commercialization of some ECL biosensing systems with high sensitivity and a wide dynamic range, such as the Elecsys technology from Roche. In this review, we provided a brief summary of the principles of ECL biosensors, and introduced common ECL luminophores, including inorganic luminophores, organic luminophores, and nanomaterials-based luminophores. The excellent properties of ECL luminophores have attracted increasing research interest as innovative emitters in ECL systems. In addition, ECL luminophores can be used not only as emitters but also donors or acceptors in ECL-RET strategies. Based on newly emerged ECL luminophores and coreactants with excellent properties, coupled with recognition molecules or multifarious signal amplification strategies, ECL biosensors have been successfully applied in the detection of mycotoxins, heavy metal ions, antibiotics, pesticide residues, foodborne pathogens, and other illegal additives.

Despite considerable progress of ECL biosensing in the past decades, several areas still need further exploration, such as screening for highly efficient luminophores, and applications in large-scale detection. Additionally, most of the biosensors reported are limited to laboratory settings and have not been commercialized. Hence, we should pay attention to the following aspects in future work:

(1) In most cases, existing biosensors could provide satisfactory sensitivity in food safety analysis, but few of them are widely used in practical applications due to analysis cost, usability, and speed. The goals of food analysis, including those of ECL biosensors and other methods, should focus on selectivity, reproducibility, and stability in complex matrixes and miniaturization of biosensors by technology; 

(2) With the development of nanotechnology, the exploration of ECL nanomaterials with the characteristics of low cost, easy synthesis, and environmental friendliness remains a future research hotspot. Currently, most nanoemitters have inferior ECL efficiency compared to Ru(bpy)_3_^2+^, but the high toxicity and poor biocompatibility of Ru(bpy)_3_^2+^ and TPrA should be considered. To fulfill ECL analysis in food matrices or biological samples, more efforts should be devoted to the development of more biocompatible, environmentally friendly, cost-effective, and efficient ECL luminophores. Additionally, most ECL emitters rely on high excitation potential, but such a high potential may cause some side effects, such as electrode passivation. Therefore, it is necessary to develop low-voltage-driven ECL emitters to improve the performance of ECL emitters; 

(3) Food matrices are complex systems that facilitate the coexistence of some pollutants, creating double toxicity and posing a threat to the environment and living organisms. Therefore, developing ECL biosensors for multiplexed detection of food contaminants is essential, and can be accomplished by developing array-based systems composed of various recognition units; 

(4) Developing sensitive analysis methods through multiple signal amplification strategies. To achieve this goal, numerous methodologies have already been employed, including DNA amplification strategies, enzyme-assisted strategies, and nanomaterials-based amplification strategies. It is important to combine these methods in a practically feasible manner for food safety analysis, and addressing these challenges will fuel the development of ECL biosensors for such analysis;

(5) Generating aptamers with high binding affinity. The screening of specific aptamers for various pollutants lays the foundation for simultaneous detection of pollutants and can promote the construction of aptasensors. Despite considerable efforts to develop ECL aptasensors, the number of aptamers is still limited. Therefore, more efforts should be devoted to aptamer screening. Currently, most work has been performed with DNA aptamers, and in order to improve biosensing performance, it is worthwhile to screen RNA aptamers; 

(6) The conjugation of biorecognition elements to the surface of nanomaterials should be sufficiently stable to avoid their release from the surface. Both aptamers and antibodies are widely used as biorecognition elements, but antibodies exhibit certain limitations. For instance, antibodies tend to be nonspecifically adsorbed onto nanomaterials, resulting in inconsistent analytical results, suggesting the necessity to protect and functionalize nanomaterials to maintain their stability and selectivity. Therefore, engineering nanomaterials and biosensor surfaces are critical for maximum ECL performance, and it is necessary to develop more strategies for more stable linkages (such as click chemistry) to produce high-quality and robust conjugates. With the rapid progress in bioconjugation chemistry, novel conjugation systems can be introduced for advanced ECL analysis with innovative principles; 

(7) Smart packaging, a future development direction in food science and technology, refers to a system used to analyze changes in packaged foods or their surroundings. ECL systems have important practical significance in accelerating the informatization and intelligentization of food analysis, playing an even greater role in promoting the effective application of active and smart packaging labels during food production;

(8) Increasing practical applications. Currently, most ECL systems are in the experimental stage of development, and the practical applications of nanomaterials-based ECL biosensors in complex samples remain a great challenge. Future research should focus on their practical applications, such as the development of stable, portable, low-cost, and user-friendly instruments for food safety analysis. Additionally, incorporating screen-printed and/or (paper-based) microfluidic systems into ECL biosensing may meet the criteria of cheap real-time detection in complex matrices. Meanwhile, developing ECL real-time assays based on mobile phones for wireless signal readout remains a substantial challenge; 

(9) Exploring new methodologies for designing ECL biosensors is still a research hotspot. Efforts should be committed to the ECL detection of single molecules, particles, and cells. ECL microscopy (ECLM) offers a platform for investigation of ECL events at the single-nanoparticle level. Limited spatiotemporal resolution is one of the potential drawbacks of ECLM, which might be associated with the low ECL efficiency of single emitters. Therefore, pursuing sensitivity down to the single-molecule level remains a research hotspot for EC biosensors.

We are optimistic that the number of nanomaterials-based ECL biosensors will increase in the future, due to ever-growing nanotechnology and our further understanding of biosensing strategies. Furthermore, commercialized ECL immunoassays will further promote the development of biomedical diagnosis, food safety analysis, and other related fields.

## Figures and Tables

**Table 3:** Brief summary of current ECL biosensors for detection of antibiotics.

Analytes	Sample Matrix	Limit of Detection	Linear Range	Ref.
amoxicillin	raw and pasteurized milk	8.3 pM	40 pM–65 μM	[69]
amoxicillin	pork, chicken, and beef	8.3 pM	50 pM to 15 nM	[70]
chloramphenicol	honey and shrimp	3.1 pg/mL	0.01–100 ng/mL	[71]
kanamycin	milk	0.36 nM	1–500 nM	[72]
kanamycin	milk	1.5 nM	10 nM–33 μM	[73]
tetracycline	pond water, milk, and honey	0.075 pg/mL	0.224–1.953 pg/mL	[74]
tetracycline	milk	32.4 nM	0.1–25 μM	[75]

## Data Availability

Not applicable.
